# Carbapenem resistance and susceptibility to Ceftazidima/avibactam and Ceftolozane/tazobactam in *Pseudomonas aeruginosa* and Enterobacterales in a reference laboratory in Lima, Peru: A cross-sectional study

**DOI:** 10.1371/journal.pone.0356004

**Published:** 2026-08-24

**Authors:** Juan Carlos Gómez de la Torre Pretell, Miguel Hueda-Zavaleta, Luis Alvarado Ríos, German Valenzuela-Rodríguez, Alberto Martin La Rosa, Thales Polis

**Affiliations:** 1 Laboratorios Roe, Lima, Lima, Peru; 2 Instituto de Investigaciones en Ciencias Biomedicas, Universidad Ricardo Palma, Lima, Lima, Peru; 3 Facultad de Ciencias de la Salud, Universidad Privada de Tacna, Tacna, Tacna, Peru; 4 MSD Peru, Lima, Lima, Peru; 5 RDMA MSD LATAM, São Paulo, São Paulo, Brazil; UAE University: United Arab Emirates University, UNITED ARAB EMIRATES

## Abstract

Carbapenem resistance in *Pseudomonas aeruginosa* and Enterobacterales is a growing threat, with intrinsic and acquired resistance mechanisms resulting in the need for new therapeutic options. In Peru, data on resistance patterns and the effectiveness of new antibiotic combinations are limited A descriptive, cross-sectional, retrospective study was conducted on *P. aeruginosa* and Enterobacterales clinical isolates collected from 2019 to 2024 at a reference laboratory in Lima, Peru, originating from various public and private healthcare centers. Bacterial identification was performed using VITEK MS MALDI-TOF, and antimicrobial susceptibility testing via VITEK 2 and/or Kirby-Bauer disk diffusion. Carbapenemase production was detected using colorimetric, immunochromatographic, and molecular methods, including the FilmArray® BCID panel for bloodstream infections. Microbiological and epidemiological variables were analyzed with Poisson regression models. A total of 173,308 isolates were analyzed, with *E. coli* (74%) and *K. pneumoniae* (10.6%) being most prevalent. Most samples (92%) were urinary. Meropenem resistance in *P. aeruginosa* reached 33% overall and 60% in respiratory specimens. *K. pneumoniae* showed 3.4% carbapenem resistance. Among carbapenem-resistant isolates, 43.7% were carbapenemase producers. Carbapenemase production was highest in carbapenem-resistant *K. pneumoniae* (93.1%), *E. coli* (87.3%), and *P. mirabilis* (77.3%), but only 32.7% in *P. aeruginosa*. Among carbapenemase-producing Enterobacterales, metallo-β-lactamases predominated, with bla-NDM detected in 69.6% of *K. pneumoniae* and bla-OXA-48-like in 100% of *E. coli*. Ceftazidime/avibactam (CZA) and ceftolozane/tazobactam (C/T) were highly active (>89%) against non-carbapenemase-producing *P. aeruginosa*, but <1% effective against producers. Resistance was more common in northern, eastern, and southern Lima. *P. aeruginosa* exhibited high carbapenem resistance, largely driven by non-carbapenemase mechanisms, supporting the role of C/T for treatment of non-carbapenemase-producing carbapenem-resistant *P. aeruginosa.* Among Enterobacterales, carbapenemase production was frequent in carbapenem-resistant isolates, with metallo-β-lactamases predominating. The limited activity of CZA against MBL producers underscores the urgent need for novel β-lactam/β-lactamase inhibitor combinations to address MBL-producing Enterobacterales.

## 1. Introduction

Antimicrobial resistance (AMR) represents one of the most urgent global public health challenges of the 21st century. It arises when microorganisms evolve mechanisms that reduce or eliminate the effectiveness of antimicrobials used to treat infections. While AMR is a natural phenomenon documented since the advent of the first antibiotics, there is growing alarm over its accelerating frequency and rapid dissemination—trends that are outpacing the development of new antimicrobial agents [1]. It is estimated that by 2050, AMR could become the leading cause of death worldwide, resulting in 10 million deaths annually and an accumulated cost of 100 trillion USD [2]. However, the Global Burden of AMR report estimated that in 2019 alone, there were 4.95 million deaths associated with, and 1.27 million deaths directly attributable to, bacterial AMR, with the highest burden observed in low- and middle-income countries [3]. These estimates position AMR as the third leading cause of death in 2019, surpassed only by ischemic heart disease and stroke [4].

Multidrug-resistant gram-negative bacteria (MDR GNB) pose a significant threat due to their inherent capacity to develop diverse resistance mechanisms—such as β-lactamase production, efflux pump activation, porin loss, and target site modifications—and to horizontally transfer resistance genes to other bacteria. These pathogens are particularly implicated in infections such as ventilator-associated pneumonia, bloodstream infections, intra-abdominal infections, and urinary tract infections [5]. Four of the six leading pathogens contributing to AMR-associated mortality are GNB: *Escherichia coli, Klebsiella pneumoniae, Acinetobacter baumannii,* and *Pseudomonas aeruginosa* [3]. A*. baumannii, P. aeruginosa*, and carbapenem-resistant Enterobacterales) were designated by the World Health Organization (WHO) as Priority 1 (Critical) pathogens for antibiotic research and development, based on criteria such as associated mortality, limited availability of effective therapies, burden of healthcare- and community-associated infections, and resistance prevalence [6]. However, according to the updated 2024 report, *P. aeruginosa* has been reclassified into the High Priority group [7].

Carbapenems play a critical role against MDR GNB; however, the prevalence of carbapenem-resistant Enterobacterales (CRE) is steadily increasing and has become endemic in various regions of the world, particularly in intensive care units (ICUs) [8]. Risk factors for CRE emergence include prior exposure to antibiotics—such as fluoroquinolones or broad-spectrum cephalosporins—and prolonged hospital stays, especially in intensive care units (ICUs) [8,9]. Patients with CRE infections have higher mortality compared to those infected with carbapenem-susceptible Enterobacterales (ENT) (RR: 2.14; 95% CI: 1.85–2.48) [10].

Most CRE isolates are carbapenemase producers (CP-CRE), belonging to class A, B, or D β-lactamases in the Ambler classification system, and account for up to 59% of CRE cases in the United States [11,12]. *Klebsiella pneumoniae* carbapenemases (KPC), which belong to Ambler class A, are the most common in the U.S., followed by New Delhi metallo-β-lactamases (NDM), Verona integron-encoded metallo-β-lactamases (VIM), and imipenem-hydrolyzing metallo-β-lactamases (IMP), all of which belong to Ambler class B, as well as oxacillinases such as OXA-48 like, classified under Ambler class D [11–13].

In Peru, carbapenem resistance has been documented in up to 16.5% of bloodstream infections caused by MDR GNB, with wide variability among species: 0% in *E. coli,* 11% in *K. pneumoniae*, 37% in *P. aeruginosa*, and as high as 60.8% in *Acinetobacter spp.* Moreover, the risk of death was significantly higher in bloodstream infections caused by *P. aeruginosa* (RR: 2.03; 95% CI: 1.09–3.78) and in those involving carbapenem-resistant (CR) strains (RR: 1.79; 95% CI: 1.05–3.06) [14].

Despite the growing threat of AMR, microbiological surveillance studies in the country remain limited, hindering the implementation of effective control and treatment strategies. Epidemiological surveillance of resistance patterns is essential to identify trends, assess the susceptibility to available antibiotics, and guide policies for the rational use of antimicrobials.

This study aims to describe the antimicrobial susceptibility patterns of *P. aeruginosa* and ENT in a reference laboratory in Lima, Peru, between 2019 and 2024, with a particular focus on carbapenem resistance and carbapenemase detection. The aim of the study is to provide data that inform clinical practice, guide antimicrobial stewardship efforts, and ultimately enhance patient outcomes.

## 2. Methods

### Study design and ethical aspects

This descriptive, cross-sectional, and retrospective study was conducted using samples with isolation of *P. aeruginosa* and Enterobacterales (ENT) processed at a reference laboratory in Lima, Peru (Laboratorios Roe), which operates 34 branches across various districts of Lima. This laboratory receives samples from both public and private healthcare institutions throughout the city.

The study period spanned from January 1, 2019, to December 31, 2024, during which a total of 185,840 positive cultures were processed. Data access and data collection were carried out between November 1, 2024, and February 28, 2025. The authors did haven't access to information that could identify individual participants during and after data collection.

The study protocol was reviewed and approved by the Vía Libre Institutional Bioethics Committee (Ethics ID: No. 11231) on October 22, 2024. Given the observational and retrospective nature of the study, and the absence of risk to participants, informed consent was not required. All data provided by the laboratory were anonymized.

### Participants

Data provided by the microbiology and informatics departments were used, containing microbiological results of samples with isolates of *P. aeruginosa* and Enterobacterales, stored in the laboratory’s database.

The study included all samples with isolates of *P. aeruginosa*, or Enterobacterales (*Escherichia coli, Klebsiella pneumoniae, Proteus mirabilis, Proteus vulgaris, Klebsiella aerogenes*, and *Enterobacter cloacae complex*) obtained from respiratory (sputum excluded), abdominal, wound/tissue, urinary, and bloodstream samples, and other samples (pleural fluid, ascitic fluid, peritoneal fluid, cerebrospinal fluid) collected from healthcare centers located in Lima (including Callao). Samples were excluded only when susceptibility results were unavailable or when polymicrobial growth was interpreted as contamination by the clinical microbiology laboratory, particularly in non-sterile specimens with no predominant pathogen. Polymicrobial cultures with a clinically significant predominant Gram-negative pathogen common in respiratory and intra-abdominal samples, were retained in the analysis.

### Procedures

**Fig 1:**
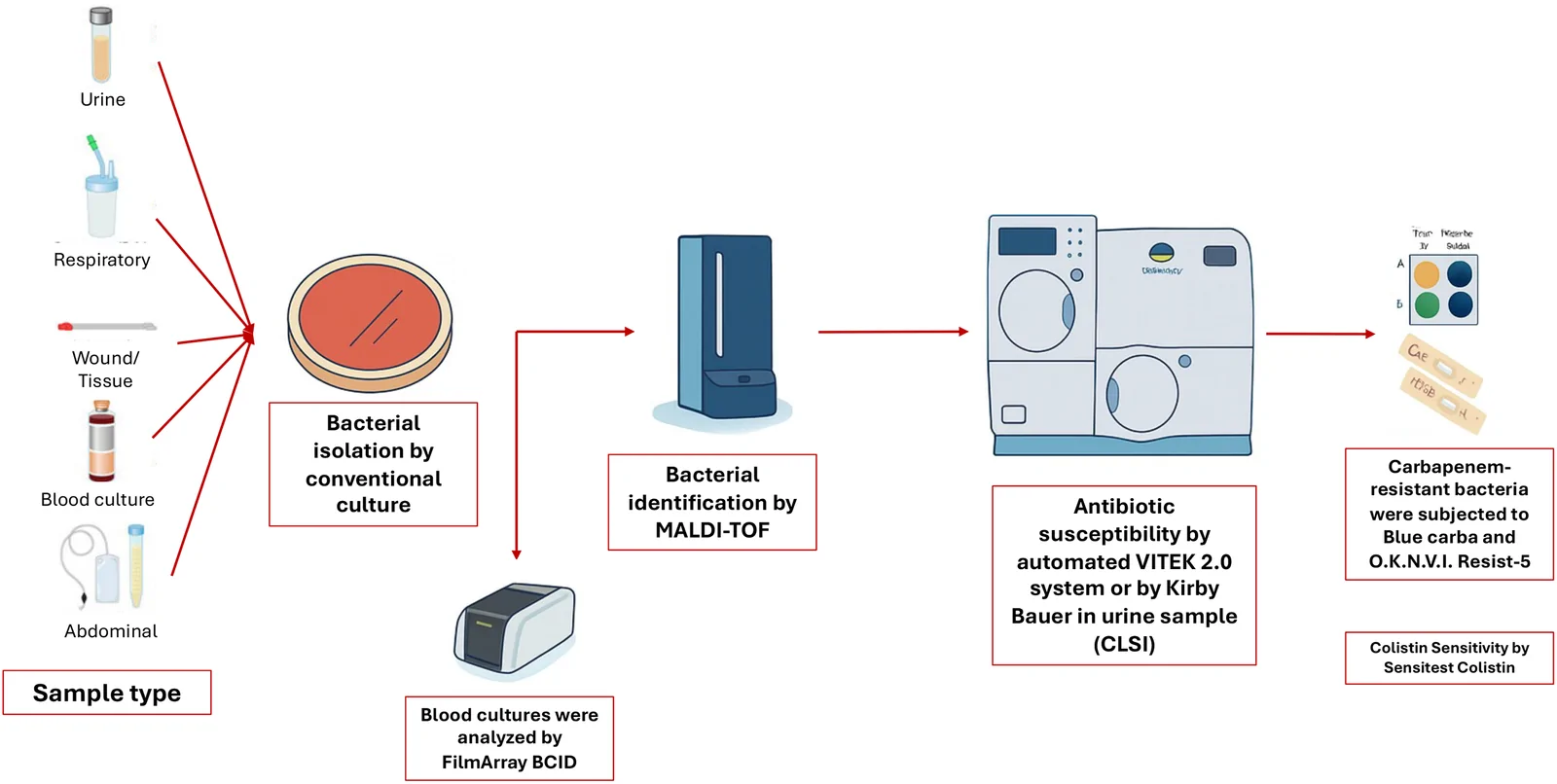
Microbiological diagnostic process and antimicrobial resistance testing in clinical samples.

Data collection was carried out by the investigators (JCGdlTP, MH-Z, and LAR) between November 2024 and February 2025. All samples underwent phenotypic susceptibility testing using automated methods (Vitek 2.0) for non-urinary specimens or disk diffusion (Kirby-Bauer) for urinary specimens, following the Clinical and Laboratory Standards Institute (CLSI) breakpoints applicable to each corresponding year. For carbapenemase detection, colorimetric methods were used (Blue Carba [ANLIS-Malbrán]), and if positive, immunochromatographic testing was performed using the OKNVI RESIST-5 (Coris Bioconcept) to identify the specific carbapenemase type. Blood culture isolates (100% of positive blood cultures are processed with molecular methods) were further analyzed using the BioFire® FilmArray® Blood Culture Identification (BCID) Panel, and from 2021 onward, the BIOFIRE® FilmArray Blood Culture Identification 2 Panel®. For colistin susceptibility testing, the Sensitest Colistin broth microdilution kit was used (Fig 1).

The variables included in the analysis were: age (in years), sex (as reported on the national identity document, with binary options: male/female), district of residence in Lima, hospitalization status (Yes/No), isolated microorganism (*P. aeruginosa, E. coli, K. pneumoniae, P. mirabilis, P. vulgaris, K. aerogenes, and Enterobacter cloacae complex*), type of specimen (blood culture, respiratory [excluding all sputum and upper respiratory tract isolates], wound/tissue, abdominal, urinary, and others samples [pleural fluid, ascitic fluid, peritoneal fluid, cerebrospinal fluid]), and antimicrobial susceptibility profile (amikacin, ciprofloxacin, piperacillin/tazobactam (TZP), imipenem, meropenem). In cases where carbapenem resistance was identified in the antibiogram, additional variables included susceptibility to CAZ), presence of carbapenemases, and type of carbapenemase detected (*bla-KPC, bla-NDM, bla-IMP, bla-VIM, bla-OXA-48-like*). For pan-drug-resistant strains, colistin susceptibility was also determined.

Additionally, the prevalence of *P. aeruginosa* with Difficult-to-Treat Resistance (DTR) was evaluated. DTR was defined as a strain resistant to at least one agent in each of the following classes: antipseudomonal β-lactams (cefepime, ceftazidime, TZP, aztreonam), carbapenems (imipenem or meropenem), and fluoroquinolones (ciprofloxacin or levofloxacin), according to the definition proposed by the IDSA guidelines [15].

### Statistical analysis

Statistical analysis was performed using Stata v17.0 (StataCorp., College Station, TX, USA) and RStudio (Version 2024.12.1 + 563, Copyright (c) 2025 Posit Software, PBC). The sample size was determined by the total number of eligible isolates available during the study period (2019–2024). Categorical variables were presented as absolute and relative frequencies, while quantitative variables were expressed as median and interquartile range (IQR) due to the non-normal distribution of the data. Proportions of susceptibility to C/T, CZA, and colistin were calculated among isolates resistant to imipenem and/or meropenem. Co-resistance and cross-resistance among antimicrobials were characterized through frequency analyses. Chi-square or Fisher’s exact tests were used, as appropriate, to compare resistance proportions according to type of healthcare system (public vs. private), age group (pediatric vs. adult), infection source (respiratory, bloodstream, urinary, wound/tissue, abdominal, others), and hospital location (ICU vs. non-ICU). Crude and adjusted prevalence ratios (PRs) were calculated using Poisson regression models with robust variance, including the same variables from the bivariate analysis as potential associated factors. A p-value < 0.05 was considered statistically significant. Additionally, a heatmap was created to illustrate the geospatial distribution of carbapenem resistance and carbapenemase prevalence by district.

## 3. Results

### Frequency of isolates and distribution by sample type

**Fig 2:**
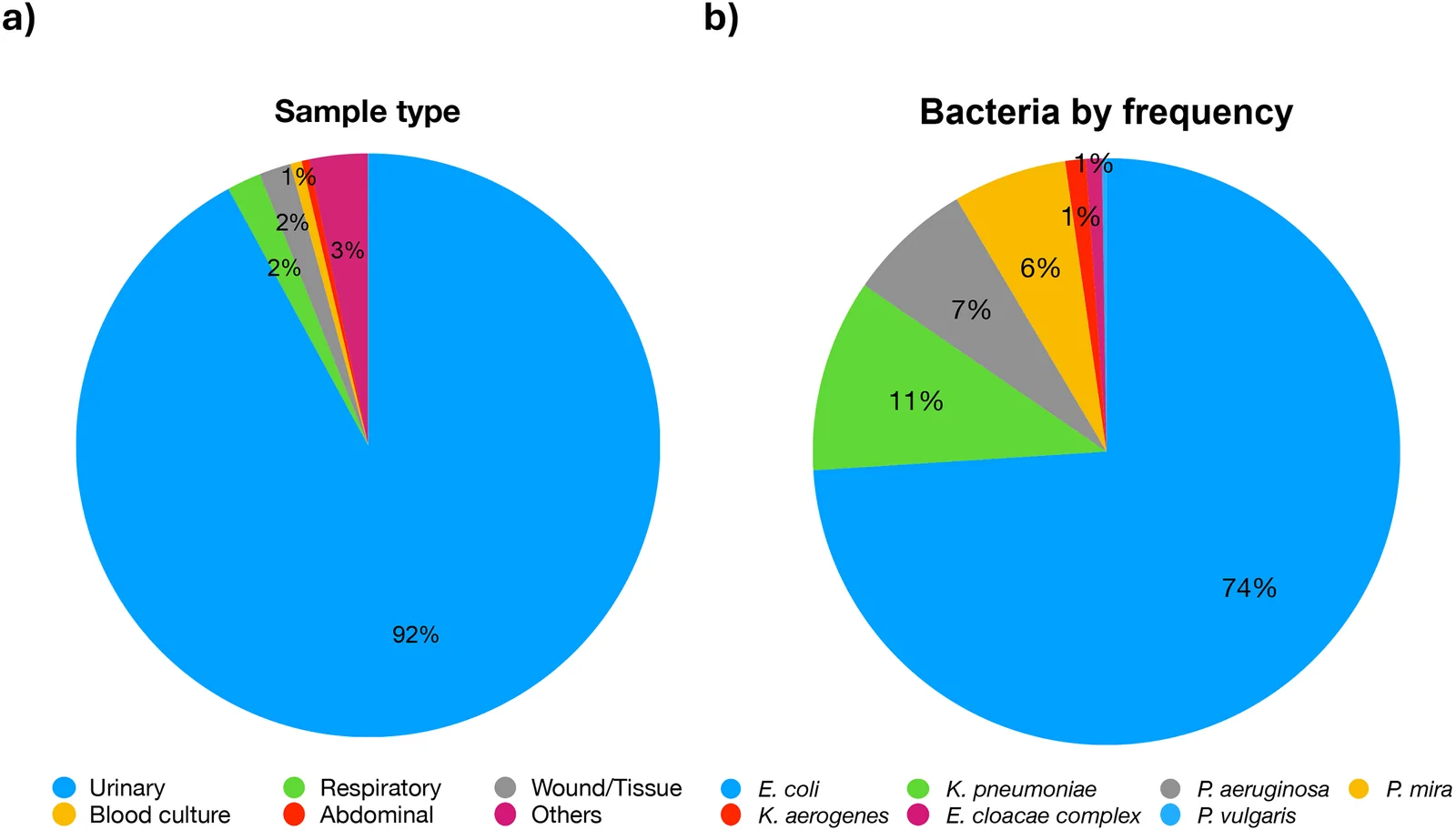
Distribution of clinical samples and isolated bacterial species. a) Distribution of clinical samples by specimen type, b) Distribution of bacterial species among all clinical isolates.

During the study period, a total of 185,840 positive cultures were processed, of which 173,308 corresponded to isolates of *P. aeruginosa*, and ENT. *Escherichia coli* was the most frequently isolated microorganism, accounting for 74% of cases (n = 128,247), followed by *Klebsiella pneumoniae* (10.6%, n = 18,358), *P. aeruginosa* (6.9%, n = 11,932), and *P. mirabilis* (6.3%, n = 10,866) (Table 1, Fig, 1b). Regarding the source of the samples, the majority were urinary (92.12%, n = 159,645), followed by respiratory samples (1.88%, n = 3,264), wound/tissue samples (1.76%, n = 3,055), and blood cultures (0.58%, n = 1,004) (Fig 2).

**Table 1:** Frequency of *Pseudomonas aeruginosa*, and Enterobacterales isolates according to sample type.

Microorganism	All samples (n = 173,308)	Blood culture (n = 1,004)	Respiratory (n = 3,264)	Wound/tissue (n = 3,055)	Abdominal(n = 725)	Urinary (n = 159,645)	Other samples (n = 5,615)
*P. aeruginosa (%)*	11,932 (6·9)	169 (15·3)	2185 (66·9)	737 (24·1)	112 (15·4)	6188 (3·9)	2541 (45·3)
*E. coli (%)*	128,247 (74·0)	445 (40·3)	262 (8·0)	1027 (33·6)	394 (54·3)	124604 (78·1)	1515 (27·0)
*K. pneumoniae (%)*	18,358 (10·6)	250 (22·6)	554 (17·0)	431 (14·1)	130 (17·9)	16057 (10·2)	936 (16·7)
*P. mirabilis (%)*	10,866 (6·3)	14 (1·3)	90 (2·8)	462 (15·1)	44 (6·1)	9941 (6·2)	315 (5·6)
*K. aerogenes (%)*	1,950 (1·1)	18 (1·6)	60 (1·8)	43 (1·4)	6 (0·8)	1759 (1·1)	64 (1·1)
*E. cloacae* ^*a*^ *(%)*	1525 (0·9)	108 (9·9)	104 (3·2)	292 (9·6)	36 (5·1)	764 (0·5)	221 (3·9)
*P. vulgaris (%)*	430 (0·2)	0 (0)	9 (0·3)	63 (2·1)	3 (0·4)	332 (0·2)	23 (0·4)

^a^E. cloacae complex. Percentages are computed within each specimen-type column (column totals = 100%)

The distribution of isolates by sample type showed that *P. aeruginosa* (67%), *K. pneumoniae* (17%), and *E. coli* (8%) were the most frequent pathogens in respiratory samples. In blood cultures, the predominant microorganisms were *E. coli* (44%), *K. pneumoniae* (25%), and *P. aeruginosa* (17%). In wound/tissue samples, the most common isolates were *E. coli* (34%), *P. aeruginosa* (24%), and *P. mirabilis* (15%). In contrast, urinary samples were dominated by ENT, with *E. coli* and *K. pneumoniae* accounting for 78% and 10% of isolates, respectively (Table 1, Fig 2).

### Antimicrobial susceptibility profile

**Fig 3:**
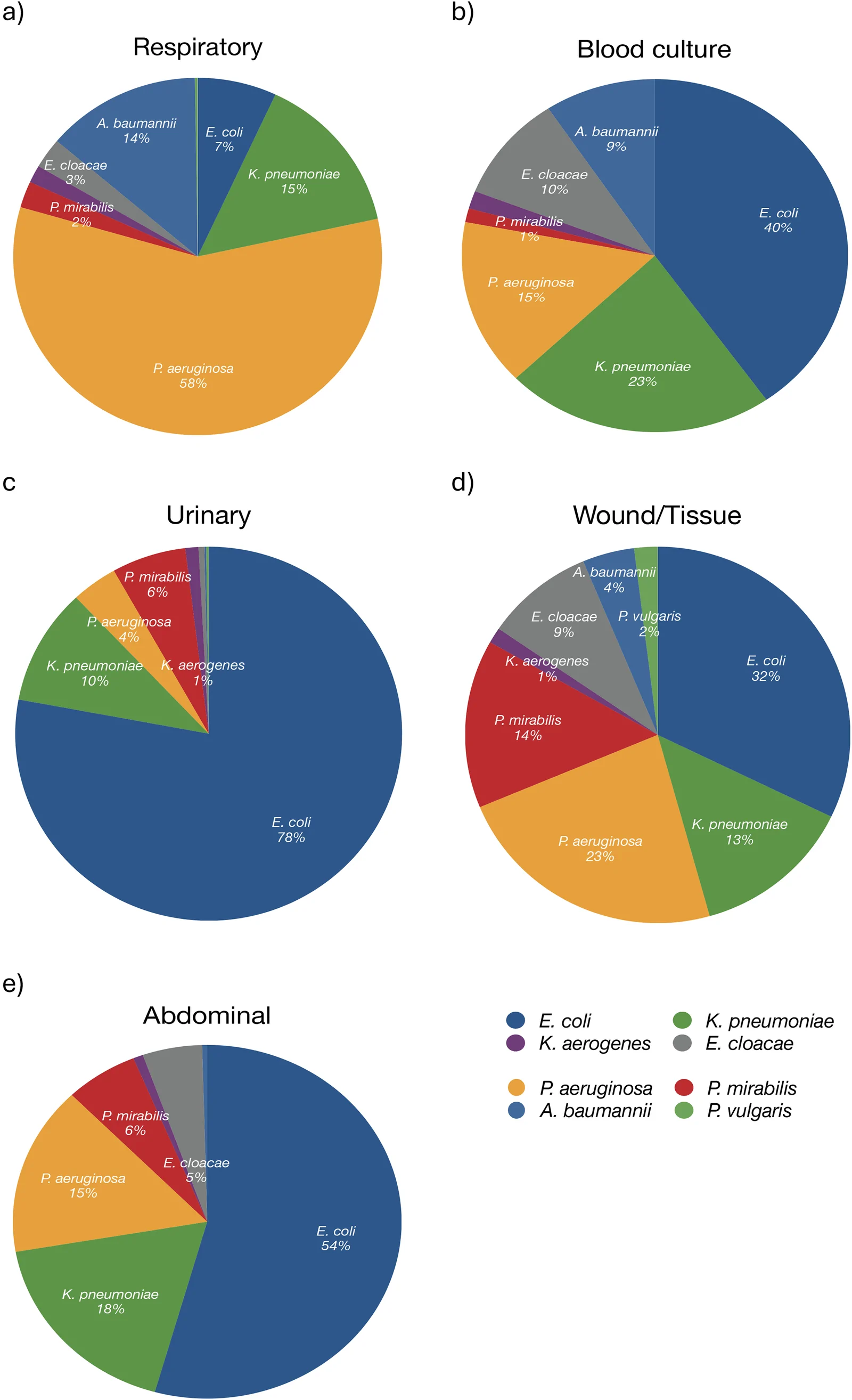
Distribution of bacterial species stratified by specimen type. a) Respiratory samples; b) Blood culture samples; c) Urinary samples; d) Wound/Tissue samples; e) abdominal samples.

Antimicrobial susceptibility varied among the different bacterial groups. Among *P. aeruginosa* isolates, the highest susceptibility rates were observed for amikacin (80.2%) and TZP (75%), while resistance to imipenem and meropenem was 34% and 33%, respectively (Table 2, Fig 3a). However, in respiratory samples and blood cultures, meropenem resistance in *P. aeruginosa* reached 60% and 38%, respectively (Fig 3b and Fig 3c). Among ENT isolates, *E. coli* showed high susceptibility to imipenem (99.8%) and meropenem (99.8%), but lower susceptibility to ciprofloxacin (39.4%), while *K. pneumoniae* presented a carbapenem resistance rate of 3.4% (Table 2, Fig 3).

**Table 2:** Antimicrobial susceptibility of *Pseudomonas aeruginosa*, and Enterobacterales isolates according to sample type.

Microorganism	Amikacin	Ciprofloxacin	Piperacillin/tazobactam	Imipenem	Meropenem
**All samples (n = 173,308)**					
*P. aeruginosa*	8,875 (80·2)	7,152 (60·2)	8,891 (75)	7,848 (65·7)	7,898 (66·2)
*E. coli*	121,700 (95)	50,493 (39·4)	121,711 (97·5)	127,781 (99·8)	127,788 (99·8)
*K. pneumoniae*	17,401 (94·8)	9,278 (50·5)	15,401 (89·6)	17,715 (96·6)	17,713 (96·6)
*P. mirabilis*	10,668 (98·3)	4,853 (44·7)	10,730 (99·1)	10,705 (99·8)	10,827 (99·8)
*K. aerogenes*	1,917 (98·4)	1,668 (85·5)	1,840 (95·9)	1,930 (99)	1,934 (99·3)
*E. cloacae* ^*a*^	1,478 (97·2)	1,058 (69·5)	1,297 (88·6)	1,483 (98)	1,492 (98·5)
*P. vulgaris*	418 (97·9)	271 (63·2)	423 (99·5)	415 (99·8)	425 (99·8)
**Blood culture (n = 1,004)**					
*P. aeruginosa*	111 (79·3)	104 (62·7)	108 (65·1)	100 (60·6)	103 (62)
*E. coli*	420 (96·8)	107 (24·7)	383 (90·3)	421 (98·1)	421 (98·1)
*K. pneumoniae*	220 (89·1)	91 (36·8)	148 (63·2)	194 (79·5)	194 (79·5)
*P. mirabilis*	13 (100)	3 (23·1)	13 (100)	11 (100)	13 (100)
*K. aerogenes*	18 (100)	17 (94·4)	14 (77·8)	15 (83·3)	15 (83·3)
*E. cloacae* ^*a*^	104 (99)	70 (66)	85 (83·3)	96 (92·3)	98 (94·2)
**Respiratory (n = 3,264)**					
*P. aeruginosa*	1,452 (74·6)	1,026 (47·7)	1,128 (52·4)	839 (39)	871 (40·4)
*E. coli*	255 (97·7)	29 (11·1)	215 (82·7)	251 (96·2)	250 (95·8)
*K. pneumoniae*	498 (89·9)	205 (37)	342 (63·5)	444 (80·3)	445 (80·5)
*P. mirabilis*	85 (94·4)	23 (25·6)	71 (78·9)	82 (98·8)	89 (98·9)
*K. aerogenes*	60 (100)	43 (71·7)	42 (71·2)	56 (93·3)	58 (96·7)
*E. cloacae* ^*a*^	99 (96·1)	81 (77·9)	83 (80·6)	101 (97·1)	101 (97·1)
*P. vulgaris*	9 (100)	6 (66·7)	8 (88·9)	7 (100)	9 (100)
**Wound/tissue (n = 3,055)**					
*P. aeruginosa*	424 (76·7)	384 (53·1)	411 (57·1)	397 (54·8)	400 (55·2)
*E. coli*	972 (96·5)	155 (15·4)	894 (90·5)	971 (97·5)	970 (97·5)
*K. pneumoniae*	410 (95·8)	129 (30·1)	271 (69·3)	379 (89·8)	379 (89·8)
*P. mirabilis*	450 (97·6)	112 (24·3)	418 (92·7)	373 (98·4)	448 (98·9)
*K. aerogenes*	42 (97·7)	29 (67·4)	36 (90)	42 (100)	42 (100)
*E. cloacae* ^*a*^	284 (97·3)	130 (44·5)	220 (80·9)	280 (96·9)	282 (97·9)
*P. vulgaris*	63 (100)	42 (66·7)	57 (98·3)	51 (98·1)	58 (98·3)
**Abdominal (n = 725)**					
*P. aeruginosa*	70 (78·7)	77 (68·8)	75 (67)	69 (61·6)	70 (62·5)
*E. coli*	370 (97·6)	87 (23)	332 (89·5)	365 (97·3)	363 (96·8)
*K. pneumoniae*	122 (95·3)	45 (35·2)	74 (62·2)	102 (81)	101 (80·2)
*P. mirabilis*	43 (97·7)	8 (18·2)	38 (86·4)	34 (97·1)	42 (95·5)
*K. aerogenes*	6 (100)	4 (66·7)	5 (83·3)	6 (100)	6 (100)
*E. cloacae* ^*a*^	36 (100)	22 (61·1)	26 (81·3)	35 (97·2)	36 (100)
*P. vulgaris*	3 (100)	3 (100)	3 (100)	3 (100)	3 (100)
**Urinary (n = 159,645)**					
*P. aeruginosa*	4,901 (79·5)	4,002 (64·7)	5,084 (82·4)	4593 (74·3)	4474 (72·3)
*E. coli*	118,247 (95)	49,690 (39·9)	118,516 (97·7)	124,291 (99·8)	124,301 (99·8)
*K. pneumoniae*	15,226 (94·9)	8,275 (51·6)	13,796 (92)	15,699 (97·8)	15,695 (97·8)
*P. mirabilis*	9,770 (98·3)	4,590 (46·2)	9,895 (99·8)	9,925 (99·9)	9,926 (99·9)
*K. aerogenes*	1,728 (98·3)	1,522 (86·5)	1,687 (97·2)	1,747 (99·4)	1,749 (99·5)
*E. cloacae* ^*a*^	739 (96·7)	630 (82·5)	702 (94)	751 (98·8)	754 (99·1)
*P. vulgaris*	321 (97·3)	203 (61·1)	332 (100)	332 (100)	332 (100)
**Other samples (n = 5,661)**					
*P. aeruginosa*	1,906 (88·2)	1,552 (61·5)	2,080 (82·5)	1,845 (73·1)	1,975 (78·3)
*E. coli*	1,434 (95·8)	424 (28·3)	1,369 (93·2)	1,480 (98·9)	1,481 (98·9)
*K. pneumoniae*	909 (97·7)	528 (56·7)	763 (85·4)	889 (95·8)	891 (96)
*P. mirabilis*	306 (98·1)	117 (37·4)	295 (96·4)	280 (100)	308 (100)
*K. aerogenes*	62 (98·4)	52 (81·3)	55 (91·7)	63 (98·4)	63 (100)
*E. cloacae* ^*a*^	214 (97·7)	125 (57·1)	180 (87·4)	218 (99·5)	219 (99·5)
*P. vulgaris*	22 (100)	17 (77·3)	23 (100)	22 (100)	23 (100)

^a^E. cloacae complex

**Fig 4:**
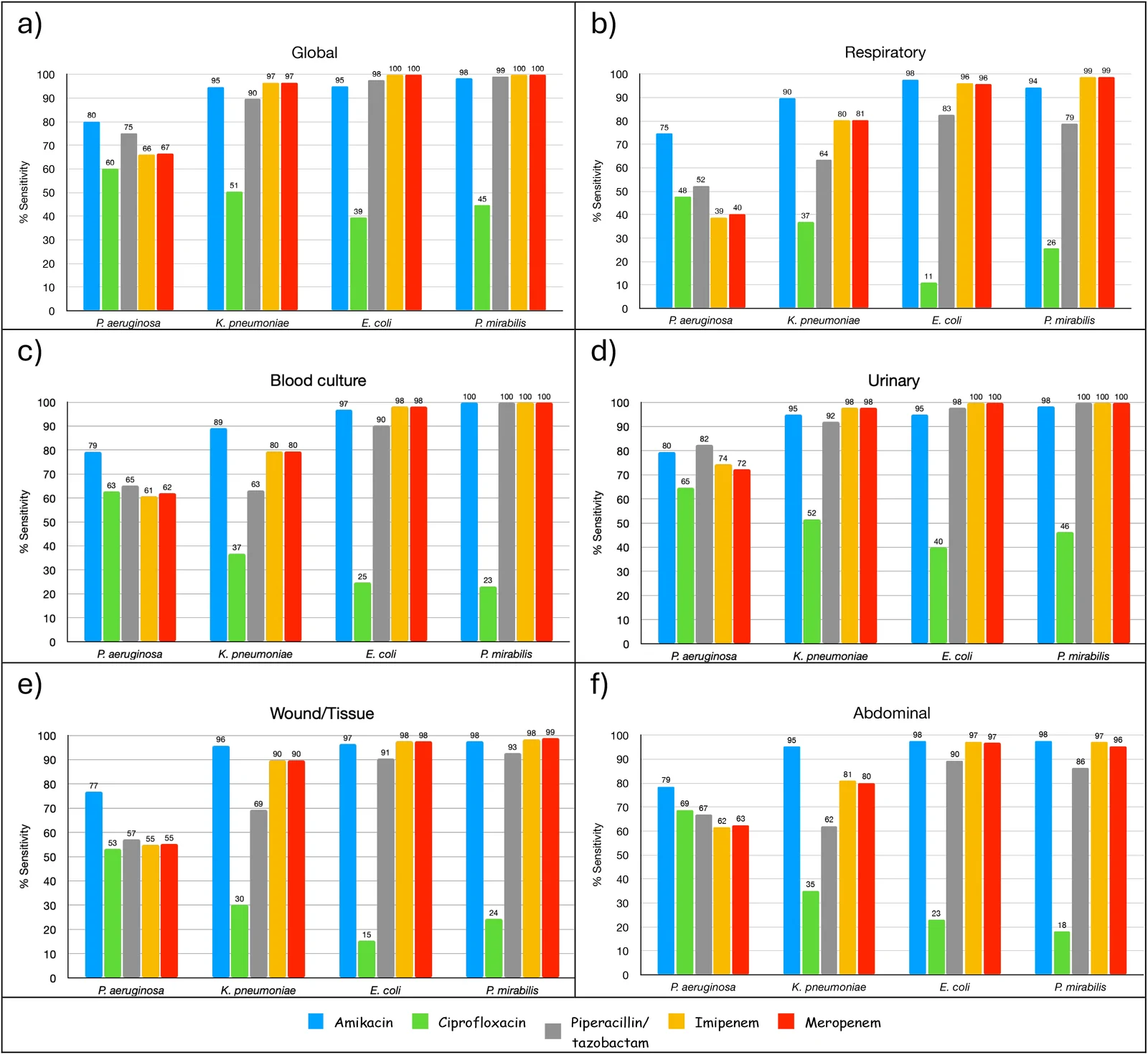
Antimicrobial susceptibility profiles of bacteria by sample type. a) Global samples; b) Respiratory samples; c) Blood culture samples; d) Urinary samples; e) Wound/tissue samples; f) Abdominal samples.

### Carbapenemase detection in carbapenem-resistant bacteria

Overall, among CR isolates (n = 4,921), 43.7% were found to be carbapenemase-producing strains (n = 2,152) (Table 3). The highest proportions of carbapenemase-producing isolates were found in CR *K. pneumoniae* (93.1%), *E. coli* (87.3%), and *P. mirabilis* (77.3%). In contrast, only 32.7% of CR *P. aeruginosa* were carbapenemase producers (Table 3). Among meropenem-resistant *P. aeruginosa* isolates from respiratory or blood samples, 26.6% were carbapenemase producers, while among meropenem-resistant *K. pneumoniae* strains, 94.90% were carbapenemase producers.

**Table 3:** Prevalence of carbapenemase production and susceptibility to Colistin, Ceftazidime/Avibactam, and Ceftolozane/Tazobactam in Isolates of *Pseudomonas aeruginosa*, and Enterobacterales with Carbapenem-Resistance, by sample type.

Microorganism	Susceptibility (%) of carbapenem-resistant isolates to each antibiotic (%)					
	Carbapenemase positive (n = 2,152/4,921)	Colistin (n = 632)	Cefepime (n = 4,870)	Piperacilina/tazobactam * (n = 4,898)	Ceftazidime/avibactam (n = 2,247)	Ceftolozane/tazobactam (n = 3,400)
**All samples (n = 4,941)**						
*P. aeruginosa (%)*	1,299/3971 (32·7)	498/528 (94·3)	1,332/3,968 (33·5)	1,413/3,959 (35·6)	1115/1957 (56·9)	1925/3400 (56·6)
*E. coli (%)*	240/275 (87·3)	11/11 (100)	5/258 (1·9)	11/272 (4·0)	82/102 (80·4)	NRT
*K. pneumoniae (%)*	573/615 (93·1)	77/92 (83·7)	5/589 (0·8)	7/608 (1·1)	105/174 (60·0)	NRT
*P. mirabilis (%)*	17/22 (77·3)	NA	1/22 (4·5)	4/21 (19·04)	1/2 (50)	NRT
*K. aerogenes (%)*	11/14 (78·5)	–	4/11 (36·3)	2/14 (14·28)	7/7 (100)	NRT
*E. cloacae*^*a*^ *(%)*	11/23 (47·8)	1/1 (100)	4/21 (19·0)	1/23 (4·3)	4/4 (100)	NRT
*P. vulgaris (%)*	1/1 (100·0)	–	0/1 (0·0)	0/1 (0·0)	0/1 (0)	NRT
**Blood culture (n = 130)**						
*P. aeruginosa (%)*	22/63 (34·9)	13/14 (92·9)	15/63 (23·8)	15/63 (23·8)	19/39 (48·7)	29/56 (51·8)
*E. coli (%)*	7/8 (87·5)	1/1 (100)	0/8 (0)	1/7 (14·3)	1/1 (100)	NRT
*K. pneumoniae (%)*	46/50 (92)	10/13 (76·9)	0/50 (0)	0/50 (0)	2/16 (12·5)	NRT
*K. aerogenes (%)*	3/3 (100)	–	0/3 (0)	0/3 (0)	3/3 (100)	NRT
*E. cloacae*^*a*^ *(%)*	0/6 (0)	.	0/6 (0)	0/6 (0)	–	NRT
**Respiratory (n = 1408)**						
*P. aeruginosa (%)*	339/1283 (26·4)	126/129 (97·7)	391/1283 (30·5)	370/1283 (28·8)	397/644 (61·6)	706/1124 (62·8)
*E. coli (%)*	9/11 (81·8)	1/1 (100)	0/11 (0)	1/11 (9·1)	4/4 (100)	NRT
*K. pneumoniae (%)*	104/108 (96·3)	16/17 (94·1)	0/108 (0)	1/108 (0·9)	3/21 (14·3)	NRT
*P. mirabilis (%)*	1/1 (100)	NA	0/1 (0)	0/1 (0)	–	NRT
*K. aerogenes (%)*	1/2 (50)	–	1/2 (50·0)	0/2 (0)	1/1 (100)	NRT
*E. cloacae*^*a*^ *(%)*	3/3 (100)	–	0/3 (0)	0/3 (0)	–	NRT
**Wound/tissue (n = 404)**						
*P. aeruginosa (%)*	109/324 (33·6)	61/68 (89·7)	86/323 (26·6)	67/320 (20·1)	105/202 (52)	163/294 (55·4)
*E. coli (%)*	21/25 (84)	2/2 (100)	0/25 (0)	1/25 (4·0)	6/10 (60)	NRT
*K. pneumoniae (%)*	42/43 (97·7)	2/3 (66·7)	0/40 (0)	0/42 (0)	2/6 (33·3)	NRT
*P. mirabilis (%)*	5/5 (100)	NA	0/5 (0)	1/5 (20·0)	1/1 (100)	NRT
*E. cloacae (%)*	6/6 (100)	–	0/5 (0)	0/6 (0)	1/1 (100)	NRT
*P. vulgaris (%)*	1/1 (100)	NA	0/1 (0)	0/1 (0)	0/1 (0)	NRT
**Abdominal (n = 81)**						
*P. aeruginosa (%)*	15/42 (35·7)	4/4 (100)	16/42 (38·1)	12/42 (28·5)	12/22 (54·5)	18/37 (48·6)
*E. coli (%)*	9/12 (75)	–	0/12 (0)	0/12 (0)	3/3 (100)	NRT
*K. pneumoniae (%)*	24/25 (96)	–	0/25 (0)	0/25 (0)	2/2 (100)	NRT
*P. mirabilis (%)*	0/2 (0)	NA	0/2 (0)	1/2 (50·0)		NRT
**Urinary (n = 2295)**						
*P. aeruginosa (%)*	690/1711 (40·3)	273/291 (93·8)	601/1709 (35.1)	730/1703 (42·8)	451/874 (51·6)	703/1424 (49·4)
*E. coli (%)*	178/202 (88·1)	6/6 (100)	5/185 (2·7)	7/200 (3·5)	63/79 (79·7)	NRT
*K. pneumoniae (%)*	322/352 (91·5)	48/57 (84·2)	5/330 (1·5)	5/346 (1·4)	94/125 (75·2)	NRT
*P. mirabilis (%)*	11/14 (78·6)	NA	1/14 (7·14)	2/13 (15·3)	0/1 (0)	NRT
*K. aerogenes*^*a*^ *(%)*	6/9 (66·7)	–	3/6 (50·0)	2/9 (22·2)	3/3 (100)	NRT
*E. cloacae (%)*	1/7 (14·3)	1/1 (100)	3/6 (50·0)	1/7 (14·3)	2/2 (100)	NRT
**Other samples (n = 603)**						
*P. aeruginosa (%)*	121/548 (22·1)	21/22 (95·5)	223/548 (40·7)	219/548 (39·9)	131/176 (74·4)	306/465 (65·8)
*E. coli (%)*	15/17 (88·2)	1/1 (100)	0/17 (0)	1/17 (5·8)	5/5 (100)	NRT
*K. pneumoniae (%)*	34/37 (91·9)	1/2 (50)	0/36 (0)	1/37 (2·7)	2/4 (50)	NRT
*E. cloacae*^*a*^ *(%)*	0/1 (0)	–	1/1 (100)	0/1 (0)	1/1 (100)	NRT

^a^E. cloacae complex; NRT: not routinely tested at laboratory; NA: not applied; * The susceptibility dose-dependent to piperacillin/tazobactam was not considered. Species-specific carbapenem-resistant denominators were used for all calculations. Testing availability differed by species and sample type. Proteus spp. is intrinsically resistant to colistin (CLSI M100, 2023).

Among 702 isolates of meropenem resistant and C/T susceptible *Pseudomonas aeruginosa* from respiratory samples, only 2 isolates (0.29%) were found to produce carbapenemases. Carbapenemase production was nearly universal among carbapenem-resistant *K. pneumoniae* (93.1%) and *E. coli* (87.3%), which translated into a substantial reduction in the activity of the studied β-lactam/β-lactamase inhibitor combinations against this subgroup.”

Molecular analysis of blood culture samples revealed that the most prevalent resistance genes among CP isolates were *bla*-NDM (69.6%) in *K. pneumoniae*, *bla*-IMP (92.3%) in *P. aeruginosa*, and *bla*-OXA-48-like (100%) in *E. coli* (Table 4). Similarly, immunochromatographic analysis across all CP *P. aeruginosa* samples showed high levels of *bla*-IMP (68.2%) and *bla*-VIM (45.5%), with co-production of both genes in 36% of cases, while CP *K. pneumoniae* showed a high prevalence of *bla*-NDM (69.6%) (Table 4).

**Table 4:** Frequency of resistance genes identified by the FilmArray Blood Culture Identification Panel (BCID) among CR *Pseudomonas aeruginosa*, and CR Enterobacterales isolated from blood cultures.

Microorganism	Carbapenemase positive (n = 39)	bla-KPC (n= 4)	bla-NDM (n= 13)	bla-IMP (n= 13)	bla-VIM (n= 7)	OXA-48 like (n = 3)
**By FilmArray Blood Culture Panel (BCID)**						
*P. aeruginosa (%)*	13/81 (16)	0/13 (0)	0/13 (0)	12/13 (92·3)	7/13 (53·8)	0/13 (0)
*E. coli (%)*	2/211 (0·9)	0/2 (0)	0/2 (0)	0/2 (0)	0/2 (0)	2/2 (100)
*K. pneumoniae (%)*	16/95 (16·8)	1/16 (6·3)	13/16 (81·3)	1/16 (6·3)	0/16 (0)	1/16 (6·3)
*K. aerogenes (%)*	3/6 (50)	3/3 (100)	0/3 (0)	0/3 (0)	0/3 (0)	0/3 (0)
**By Immunochromatography**						
**Microorganism**	**Carbapenemase positive(n = 78)**	**KPC(n = 5)**	**NDM(n = 37)**	**IMP(n = 16)**	**VIM(n = 10)**	**OXA-48 Like(n = 3)**
*P. aeruginosa (%)*	22/169 (13)	0/22 (0)	0/22 (0)	15/22 (68·2)	10/22 (45·5)	0/22 (0)
*E. coli (%)*	7/445 (1·6)	0/7 (0)	5/7 (71·4)	0/7 (0)	0/7 (0)	2/7 (28·6)
K. pneumoniae (%)*	46/250 (18·4)	2/46 (4·3)	32/46 (69·6)	1/46 (2·2)	0/46 (0)	1/46 (2·2)
*K. aerogenes (%)*	3/18 (16·7)	3/3 (100)	0/3 (0)	0/3 (0)	0/3 (0)	0/3 (0)

bla-KPC: *Klebsiella pneumoniae* carbapenemase; NDM: New Delhi metallo-β-lactamases; VIM: Verona integron-encoded metallo-β-lactamases; IMP: imipenem-hydrolyzing metallo-β-lactamases; *OXA-48* Like: oxacillinase *OXA-48* family. * Ten patients with *K. pneumoniae* isolated with carbapenemase positive by immunochromatography were unaffiliated. Testing availability differed by species and sample type.

### Antibiotic susceptibility in outpatients and hospitalized patients

Isolates from hospitalized patients exhibited higher resistance rates to ciprofloxacin (60.4% vs. 56.0%), cefepime (45.5% vs. 37.3%), and meropenem (8.9% vs. 2.3%) compared to those from non-hospitalized patients (Fig 4a).

In the subgroup analysis of *P. aeruginosa* (Fig 4b), hospital-acquired isolates showed greater resistance to ciprofloxacin (43.0% vs. 38.0%), meropenem (48.6% vs. 29.7%), and imipenem (48.2% vs. 29.7%). The prevalence of DTR phenotypes was also higher in hospital isolates (27.3% vs. 17.3%).

### Therapeutic options for carbapenem-resistant microorganisms

**Fig 5:**
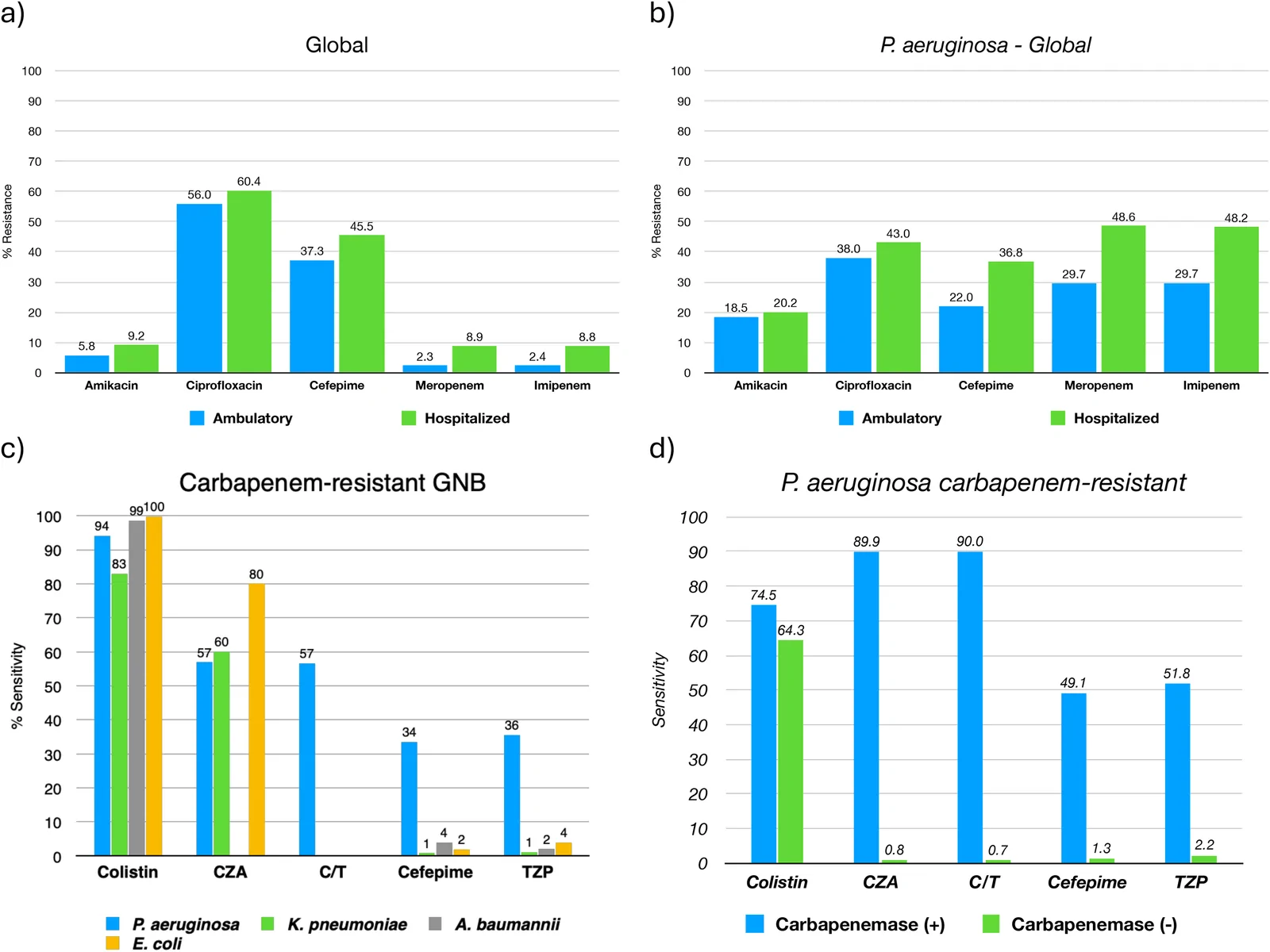
Antibiotic resistance and susceptibility profiles among ambulatory and hospitalized patients with Gram-negative bacterial isolates. a) Overall prevalence of antibiotic resistance among ambulatory and hospitalized patients. b) Prevalence of antibiotic resistance in *Pseudomonas aeruginosa* isolates from ambulatory and hospitalized patients. c) Antibiotic susceptibility profiles of carbapenem-resistant Gram-negative bacteria. d) Antibiotic susceptibility of carbapenem-resistant *Pseudomonas aeruginosa* according to carbapenemase production status. Abbreviations: DTR, difficult-to-treat resistance; CZA, ceftazidime-avibactam; C/T, ceftolozane-tazobactam; TZP, piperacillin-tazobactam.

Among CR isolates, susceptibility to colistin, CZA, and C/T varied. Colistin demonstrated the highest activity against CR *E. coli* and *P. aeruginosa*, with susceptibility rates exceeding 90%. In contrast, only 83% of CR K*. pneumoniae* isolates were susceptible to colistin. In the subgroup analysis of CR *P. aeruginosa*, susceptibility was 94.3% to colistin, 56.9% to CZA, and 56.6% to C/T (Fig 5c). Susceptibility to C/T varied by infection source, with superior activity in respiratory samples (62.8%) compared to intra-abdominal samples (48.6%), whilst CZA showed more consistent activity across different sites. Among CR *P. aeruginosa isolates*, those without detectable carbapenemases showed high susceptibility to CZA (89.9%) and C/T (90%). In contrast, when carbapenemases were present, susceptibility dropped to <1% for both agents (Fig 5d).

Among ENT, *E. coli* and *K. pneumoniae* showed susceptibility rates of 80.4% and 60%, respectively, to CZA (Table 3). In summary, approximately one-third of *P. aeruginosa* isolates were resistant to meropenem, with the highest rates observed in respiratory specimens. Among carbapenem-resistant isolates, carbapenemase production was uncommon, and both C/T and CZA retained activity against the majority of non-carbapenemase-producing isolates.

### Geographic distribution of carbapenem resistance in lima

Elevated levels of bacterial meropenem-resistance were observed in districts such as San Juan de Lurigancho, San Martín de Porres, and Ate, exceeding 50% in some cases. In contrast, districts like Miraflores, San Borja, and La Molina were found to have significantly lower resistance rates, below 20%. This geographic variability highlights potential differences in antimicrobial pressure or local infection control practices. Carbapenemase-producing bacteria were particularly prevalent in the northern and eastern districts of Lima, reaching proportions above 30% in some areas. In the geographic distribution of carbapenemase-producing *P. aeruginosa*, higher prevalences were observed in southern districts (Punta Negra, Punta Hermosa) and northern districts (Independencia, Santa Rosa), with rates exceeding 50%. In contrast, most central and eastern districts exhibited considerably lower proportions, reflecting a heterogeneous distribution pattern.

### Factors associated with carbapenem resistance

A total of 81% of patients were female, with a media age of 64.8 years (SD: 24.74); 58.8% were older than 64 years, and only 7.7% of patients were reported as hospitalized. Carbapenem resistance was more prevalent among male patients, hospitalized individuals, and isolates from blood cultures, respiratory, and abdominal samples (Table 5).

**Table 5:** Clinical and microbiological characteristics associated with carbapenem resistance and carbapenemase production in *P. aeruginosa* and Enterobacterales isolates.

Variable	Total	Carbapenem resistance		p-value
		Yes	No	
Sex (n = 133,831)				**<0**·**001**^**a**^
• Female	109,079 (81·50)	1,431 (1·3)	107,648 (98·7)	
• Male	24,752 (18·50)	2,275 (9·2)	22,477 (90·8)	
Age* (SD)	64.84 (24·74)	68·57 (21.04)	64.73 (24.83)	**<0**·**001**^**b**^
Age categorized (n = 134,607)				**<0**·**001** ^**a**^
• Neonate	452 (0·33)	2 (0·4)	450 (99·6)	
• 1–5 years	4,816 (3·57)	22 (0·5)	4,794 (99·5)	
• 6–18 years	3,942 (2·93)	78 (1·9)	3,864 (98·1)	
• 19–44 years	17,130 (12·73)	388 (2·27)	16,742 (97·73)	•
• 45–64 years	29,088 (21·62)	850 (2·92)	28,238 (97·08)	
• ≥ 65 years	79,179 (58·82)	2,351 (2·97)	76,828 (97·03)	
Hospitalized (n = 137,350)				**<0**·**001** ^**a**^
• Yes	10,640 (7·75)	943 (8·86)	9,697 (91·14)	
• No	126,710 (92·25)	2,969 (2·34)	123,741 (97·66)	
Sample type (n = 172,979)				**<0**·**001**^**a**^
• Abdominal	702 (0·40)	81 (11·54)	621 (88·46)	
• Blood culture	974 (0·56)	130 (13·35)	844 (86·65)	
• Wound /tissue	2,983 (1·73)	404 (13·54)	2,579 (86·46)	
• Respiratory	3,231 (1·87)	1,408 (43·58)	1,823 (56·42)	
• Urine	159,526 (92·22)	2,295 (1·44)	157,231 (98·56)	
• Others	5,563 (3·22)	603 (10·84)	4,960 (89·16)	

^a^ = Chi^2^; ^b^ = Two-sample T test Student; * median; SD = standard deviation

Multivariate analysis showed that male sex (aPR: 3·34; 95% CI: 3·07–3·63), older age, and hospitalization (aPR: 1·25; 95% CI: 1·21–1·29) were significantly associated with a higher prevalence of carbapenem resistance. Additionally, compared to urinary samples, respiratory (aPR: 15·35; 95% CI: 14·01–16·83), blood culture (aPR: 5·46; 95% CI: 4·47–6·67), and wound/tissue samples (aPR: 5·52; 95% CI: 4·86–6·27) showed significantly higher prevalence ratios, indicating that both sample type and clinical context influence observed resistance levels (Table 6).

**Table 6:** Prevalence ratios of carbapenem resistance in Gram-negative bacteria.

Variable	cPR (95% CI)	p value	aPR (95% CI)	p value
Sex				
• Female	*Reference*		*Reference*	
• Male	7·00 (6·56–7·47)	<0·001	3.34 (3.07–3.63)	**<0**·**001**
Age	1·006 (1·005–1·007)	<0·001	1·25 (1·21–1·29)	**<0**·**001**
Age categorized (%)				
• Neonate	*Reference*		–	
• 1–5 years	1·03 (0·24–4·37)	0·965		
• 6–18 years	4·47 (1·10–18·13)	0·036		
• 19–44 years	5·11 (1·27–20·47)	0·021		
• 45–64 years	6·60 (1·65–26·36)	0·008		
• ≥ 65 years	6·71 (1·68–26·76)	0·007		
Hospitalized				
• No	*Reference*		*Reference*	
• Yes	3·79 (3·53–4·07)	<0·001	1.52 (1.41–1.65)	**<0.001**
Sample type				
• Urine	*Reference*		*Reference*	
• Blood culture	12·96 (11·41–14·73)	<0·001	5·46 (4·47–6·67)	**<0**·**001**
• Abdominal	8·02 (6·50–9·88)	<0·001	4·23 (3·17–5·66)	**<0**·**001**
• Wound/tissue	9·41 (8·52–10·39)	<0·001	5·52 (4·86–6·27)	**<0**·**001**
• Respiratory	30·29 (28·62–32·05)	<0·001	15·35 (14·01–16·83)	**<0**·**001**
• Others	7·53 (6·91–8·20)	<0·001	4·92 (4·39–5·52)	**<0**·**001**

cPR: crude Prevalence Ratio; aPR: Adjusted Prevalence Ratio; 95% CI: 95 percent confidence interval

### Temporal trends of carbapenem resistance

**Fig 6:**
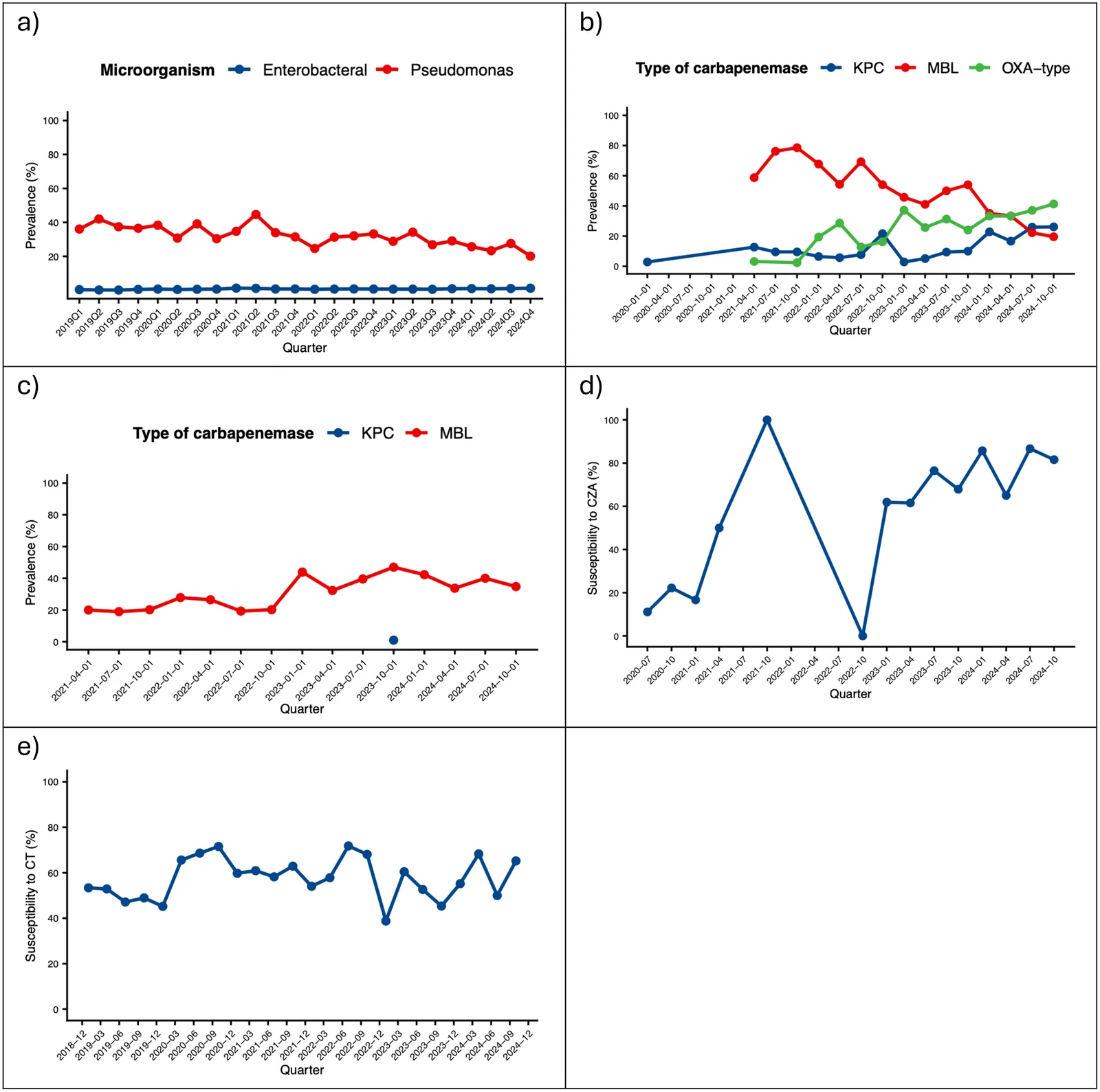
Temporal trends (2019–2024, overall) in carbapenem resistance, carbapenemase types, and β-lactam/β-lactamase–inhibitor susceptibility in *P. aeruginosa* and Enterobacterales: (a) carbapenem resistance in *P. aeruginosa* and Enterobacterales; (b) Carbapenemase types in carbapenem-resistant Enterobacterales; (c) Carbapenemase types in carbapenem-resistant *P. aeruginosa*; (d) Ceftazidime/avibactam susceptibility in carbapenem-resistant Enterobacterales; (e) Ceftolozane/tazobactam susceptibility in carbapenem-resistant *P. aeruginosa*; CZA: Ceftazidime/avibactam; CT: Ceftolozane/tazobactam.

Over 2019–2024, including all samples, the prevalence of carbapenem resistance was consistently higher in *P. aeruginosa* than in ENT, with the latter remaining low and relatively stable. Among CRE, carbapenemase types were dominated by MBL, while OXA-48 like increased toward the later quarters and KPC remained low. In CRPA, the distribution was almost exclusively MBL, with KPC only sporadically observed. CZA susceptibility in CRE showed an overall upward trend, reaching high proportions in 2022–2024 with quarter-to-quarter fluctuations. In CRPA, C/T susceptibility remained in intermediate ranges with temporal variability, and respiratory samples mirrored the overall cohort without sustained differences (Fig 6).

## 4. Discussion

In this study, which included over 170,000 clinical isolates from Lima, Peru, we observed a high burden of AMR in *P. aeruginosa* and ENT. *Escherichia coli* was the most frequently isolated microorganism, followed by *K. pneumoniae* and *P. aeruginosa*. A high overall prevalence of carbapenem resistance was observed in *P. aeruginosa* (33%), while resistance among ENT was overall lower (<2%). Notably, despite the high frequency of CRPA, carbapenemase production was less prevalent (32.7%) compared to ENT, where a large proportion of carbapenem-resistant strains were carbapenemase producers (93.1% in *K. pneumoniae* and 87.3% in *E. coli*). This suggests that non-carbapenemase mechanisms, such as OprD porin loss and efflux pump overexpression, predominantly drive carbapenem resistance in *P. aeruginosa.* Among CRE isolates, the predominant resistance mechanisms were the bla-NDM, bla-IMP, and bla-VIM genes. Moreover, we identified marked geographic variability in the distribution of resistance across Lima, with critical hotspots in the northern and eastern districts. These results reinforce the need to strengthen local microbiological surveillance and optimize antimicrobial use in clinical practice.

Among *Pseudomonas aeruginosa* isolates, overall susceptibility to C/T was 84.9%, and 56.6% among carbapenem-resistant (CR) isolates. Higher susceptibility was observed in isolates from respiratory infections (62.8%) compared to those from intra-abdominal infections (48.6%). These findings highlight the importance of considering both the type of microorganism and the site of infection when selecting C/T as a therapeutic option, underscoring its value in treating respiratory infections caused by non-carbapenemase producing carbapenem resistant *P. aeruginosa* (NCP-CRPA). Furthermore, by applying two carbapenemase-detection assays to carbapenem-resistant isolates, we determined that almost all meropenem-resistant but C/T-susceptible *Pseudomonas aeruginosa* isolates were non-carbapenemase producers, supporting that C/T susceptibility may be a practical surrogate marker for absence of carbapenemases.

In our study, blood culture isolates showed a relatively favorable susceptibility profile for *E. coli* and *K. pneumoniae*, with meropenem resistance rates of 1.9% and 20.5%, respectively. These values are lower than those reported by the WHO GLASS 2022 surveillance for bloodstream infections in the Americas, where meropenem resistance was 1.3% in *E. coli* and 29.4% in *K. pneumoniae* [16]. Our findings are also consistent with the global multicenter study by Sader et al. (2021), which reported a global carbapenem resistance rate of 4.5% in CRE collected from 64 centers worldwide [17]. These results align with the report by Han et al. (2017), who documented high rates of carbapenem resistance in *Klebsiella spp*. (up to 24.6%) in long-term care hospitals in the United States, highlighting a shared regional and global threat [16,18]. Similarly, a recent multicenter study in Peru confirmed this concerning trend, reporting high resistance rates in bloodstream infections caused by GNB: 59.2% for third-generation cephalosporins, 16.5% for carbapenems, and 63.5% for fluoroquinolones. Specifically, carbapenem resistance was 60.8% in *Acinetobacter spp*., 37% in *P. aeruginosa*, 11% in *K. pneumoniae*, and 0% in *E. coli* [14]. These figures are particularly relevant considering that resistance to fluoroquinolones and β-lactam antibiotics (including carbapenems, cephalosporins, and penicillin) accounted for over 70% of all AMR-attributable deaths across pathogens, according to recent global estimates [3].

Regarding *P. aeruginosa*, we observed a pattern of high AMR, particularly to carbapenems. In our isolates, meropenem resistance in *P. aeruginosa* was 33%, reaching 59.6% in respiratory samples—higher than the rate reported by the CDC/NHSN in the United States (25.8%) and above the average rates described in low- and middle-income countries, where resistance to imipenem reaches 50.7% and to ceftazidime 45% [19]. This disparity suggests a critical resistance scenario in the Peruvian context, likely driven by inappropriate antibiotic use and the limited implementation of antimicrobial stewardship programs. This high level of resistance also aligns with data from the WHO GLASS study in Latin America (71.5%) and with findings from Han et al. in the U.S. in long-term care settings [16,18].

Likewise, the presence of carbapenemases in *P. aeruginosa* varies significantly by region. While a multinational study reported that only 2% of CR *P. aeruginosa* isolates in the United States harbored these enzymes, the proportion reached 69% in Central and South America and 57% in Australia and Singapore (20). In our findings, 32.7% of CR *P. aeruginosa* isolates were carbapenemase producers (26.6% among meropenem resistant isolates from respiratory samples or blood), in contrast to *Klebsiella pneumoniae*, where 93.1% of CR strains carried carbapenemases. This genetic difference underscores the need for local molecular studies to better characterize resistance mechanisms in our setting, which could inform more targeted control strategies and improve antimicrobial treatment selection.

In line with regional analyses showing a growing trend of ENT carrying NDM, our *K. pneumoniae* isolates demonstrated a clear predominance of this gene. Although KPC remains an important carbapenemase in some countries, its frequency in our study was low, consistent with findings from other areas of Latin America and particularly in the MEA region (Middle East and Africa), where OXA-48 like is more prevalent [20]. In CP *P. aeruginosa*, the detection of a significant proportion of IMP and VIM genes, and the absence of KPC and OXA-48 like, closely aligns with previous local reports. Consistent with our findings, previous local reports from Peru have documented the predominance of IMP and VIM among carbapenemase-producing P. aeruginosa [21], as well as additional carbapenemase variants in Enterobacterales [22,23], reinforcing the heterogeneity of resistance mechanisms in the region.

In our study, the susceptibility rates to CZA and C/T were comparable, even among CR bacteria. Notably, *P. aeruginosa* isolates that were resistant to carbapenems but lacked detectable carbapenemases showed a 90% susceptibility rate to both C/T and CZA. According to the IDSA guidelines, preferred therapy for infections caused by difficult-to-treat resistant (DTR) *P. aeruginosa* includes C/T, CZA and imipenem-cilastatin-relebactam, particularly for cystitis, pyelonephritis, and extra-urinary infections. However, the guidelines do not favor one agent over another due to insufficient clinical trial data at the time of publication [15]. Treatment selection should align with local susceptibility profiles and the underlying resistance mechanism. Current international guidelines—including the IDSA 2024 recommendations [15] and recent Latin American consensus documents [24] generally support a mechanism-based approach in which agents active against AmpC- and OprD-mediated resistance may be considered for non-carbapenemase-producing isolates, while agents with activity against serine carbapenemases are reserved for carbapenemase-producing or difficult-to-treat resistant strains. The relative merits of each β-lactam/β-lactamase inhibitor combination remain a matter of ongoing clinical debate.

Several studies have compared the clinical outcomes of both antibiotics, finding no statistically significant differences in mortality (44% vs. 37%, p = 0.31), clinical cure (61% vs. 66%, p = 0.46), or adverse events such as kidney injury (23% vs. 17%, p = 0.28). However, resistance emergence appeared more frequently in the C/T group (38% vs. 25%, p = 0.09) [25,26]. These studies presented some limitations that may have introduced bias—for example, borderline susceptibility was more common in the C/T group (59%) than in the CZA group (25%), and 20% of patients receiving C/T did not receive an appropriate dose, which could have influenced outcomes.

More recent comparative evidence has yielded mixed results. The CACTUS multicenter retrospective study reported a higher likelihood of clinical cure with CT compared to CZA in multidrug-resistant P. aeruginosa infections (61% vs. 52%; aOR 2.07), particularly in pneumonia cases (63% vs. 51%; aOR 2.34), with no significant differences in mortality, hospital stay, or duration of mechanical ventilation [27]. These results contrast with prior studies that found no clinical differences between agents [25,26]; the discrepancy has been variously attributed to differences in the study population (proportion of urinary versus respiratory infections), CT dosing, and the extent of susceptibility testing performed. Beyond clinical outcomes, the two agents have distinct mechanistic profiles: ceftolozane is less susceptible to hydrolysis by AmpC enzymes and less affected by porin loss, while avibactam binds covalently and reversibly to β-lactamases and uniquely inhibits class A and OXA-48-like carbapenemases—an ability lacking in tazobactam [28]. Together, these data suggest that the choice between CZA and CT should be guided by the local mechanism of resistance and individual patient factors rather than by an assumed superiority of either agent.

Our findings demonstrate distinct carbapenem-resistance patterns driven by fundamental differences in resistance mechanisms across pathogens. *P. aeruginosa* showed persistently high resistance rates (30–50%), contrasting with the stable low levels in ENT (1–3%), reflecting the intrinsic and adaptive complexity of *P. aeruginosa* resistance pathways [6,7]. CP-CRPA was almost exclusively mediated by metallo-β-lactamases (>95%), consistent with prior Peruvian reports identifying MBL as the dominant mechanism in high-risk lineages [21], unlike regions where KPC predominates. In CRE, MBLs were also predominant (60–90%), accompanied by a progressive rise in OXA-type carbapenemases (10% → 40%), reflecting the global evolution toward more diverse resistance mechanisms with major therapeutic implications [20]. CZA maintained high activity against CRE (>80% in multiple quarters from 2022–2024), while C/T exhibited moderate and variable susceptibility in CRPA (40–70%), consistent with its limited efficacy against MBL-producing strains [24,27,28]. These trends underscore the need for therapeutic strategies targeting MBL-producing organisms, including novel combinations such as aztreonam/avibactam (ATM-AVI), which has demonstrated promising activity against MBL-producing ENT [29,30].

Comparative studies of C/T versus polymyxins or aminoglycosides have shown higher clinical cure rates (aOR: 2.63) and lower prevalence of acute kidney injury (AKI) (aOR: 0.08) with CT, although no significant difference was observed in in-hospital mortality (34). This may be explained by the poor pharmacokinetics, narrow therapeutic index, and high nephrotoxicity rates associated with polymyxins and aminoglycosides. A meta-analysis reported nephrotoxicity rates of 29.8% for polymyxin B and 26.7% for colistin [31], which may represent an unacceptably high toxicity burden—particularly in critically ill patients.

The high prevalence of metallo-β-lactamases (MBLs) in our setting represents a significant therapeutic challenge due to their characteristic multidrug-resistant phenotype and the limited treatment options available. The IDSA guidelines recommend, as the preferred strategy for infections caused by MBL-producing ENT, the use of CZA in combination with aztreonam or, alternatively, monotherapy with cefiderocol [15]. These recommendations are based on extrapolated estimates from the ATM-AVI combination, which is currently not commercially available in the United States. Two observational studies have evaluated treatment options for infections caused by MBL-producing ENT, predominantly NDM producers. In the first study, the combination of CZA with aztreonam was associated with a lower 30-day mortality rate (19%) compared to other therapies (44%) [32]. In the second study, this combination also showed reduced mortality (22%) compared to cefiderocol (33%) and colistin (50%), confirming its association with better clinical outcomes over colistin-based regimens, even among patients with bacteremia [30].

ATM-AVI stands out for its stability against all four Ambler classes of β-lactamases, making it a versatile option against multidrug-resistant GNB (34). It has demonstrated higher antimicrobial activity against approximately 80–85% of MBL-producing ENT compared to CZA or aztreonam monotherapy. However, the addition of avibactam to aztreonam did not restore susceptibility in isolates of *P. aeruginosa* [33], and only 6% of MBL-producing Pseudomonas isolates were susceptible to ATM-AVI [34,35]. This suggests that aztreonam resistance in these species is primarily driven by other mechanisms, such as efflux pump overexpression, membrane porin loss, and the presence of OXA-type enzyme variants.

In our series, a marked geographic variability was observed in the prevalence of CR and carbapenemase-producing bacteria across districts of Lima, Peru. Districts such as San Juan de Lurigancho, San Martín de Porres, Ate, Independencia, and Punta Hermosa showed the highest proportions, in some cases exceeding 50%. This pattern may be related to the proximity of large public hospitals or referral centers located in these areas, which often receive more complex patients with prior exposure to broad-spectrum antibiotics—factors that favor selective pressure and the dissemination of multidrug-resistant strains.

This study has several limitations. First, being an observational and retrospective review of a microbiological data base, there was limited access to detailed clinical information, preventing correlation of microbiological findings with clinical outcomes such as treatment response or mortality. In addition, because the study was conducted using data from a reference laboratory that receives samples from multiple institutions with varying levels of complexity, it was not possible to standardize the clinical context or the sampling methods. Furthermore, although molecular and immunochromatographic methods were used for carbapenemase detection, these do not allow full characterization of resistance mechanisms, which limits epidemiological interpretation. Lastly, since the majority of samples originated from outpatient settings and private healthcare facilities, the results may not fully reflect the situation in high-complexity public hospitals in other regions of the country. This could potentially introduce selection bias, inclusion bias, and access-to-care bias. Prospective, multicenter studies that incorporate clinical data and allow for the correlation of microbiological profiles with relevant clinical outcomes are strongly recommended.

## 5. Conclusion

*P. aeruginosa* in Lima exhibited high carbapenem resistance, especially in respiratory and nosocomial contexts, largely driven by non-carbapenemase mechanisms. This cautions against empiric carbapenem use for suspected *P. aeruginosa* infection and, in line with current clinical guidelines, our findings are consistent with the use of β-lactam/β-lactamase inhibitor combinations selected according to the underlying resistance mechanism — particularly the presence or absence of carbapenemase production, and according to local susceptibility patterns. In contrast, carbapenem resistance was relatively less common among Enterobacterales, however, carbapenemase production was frequent among CRE isolates. While CZA demonstrates activity against KPC and OXA-48-like producers, it lacks activity against metallo-β-lactamases. The predominance of MBLs among carbapenemase producers, with only ~60% susceptibility to CZA, underscores the urgent need for novel β-lactam/β-lactamase inhibitor combinations and alternative therapeutic strategies to address the growing burden of MBL-producing Enterobacterales.

## Supporting information

S1 Appendix List of abbreviations. https://doi.org/10.6084/m9.figshare.32413185
(DOCX)

S2 Dataset Anonymized dataset used for the study analyses. https://doi.org/10.6084/m9.figshare.33155150
(ZIP)
