# Correction: Carbapenem resistance and susceptibility to Ceftazidima/avibactam and Ceftolozane/tazobactam in *Pseudomonas aeruginosa* and Enterobacterales in a reference laboratory in Lima, Peru: A cross-sectional study

**DOI:** 10.1371/journal.pone.0362811

**Published:** 2026-11-11

**Authors:** Juan Carlos Gómez de la Torre Pretell, Miguel Hueda-Zavaleta, Luis Alvarado Ríos, German Valenzuela-Rodríguez, Alberto Martin La Rosa, Thales Polis

In Fig 5, there is an error in panel D. The susceptibility values were inadvertently assigned to the opposite carbapenemase-production categories in the graphical representation. Please see the correct Fig 5 here.

**Fig 5:**
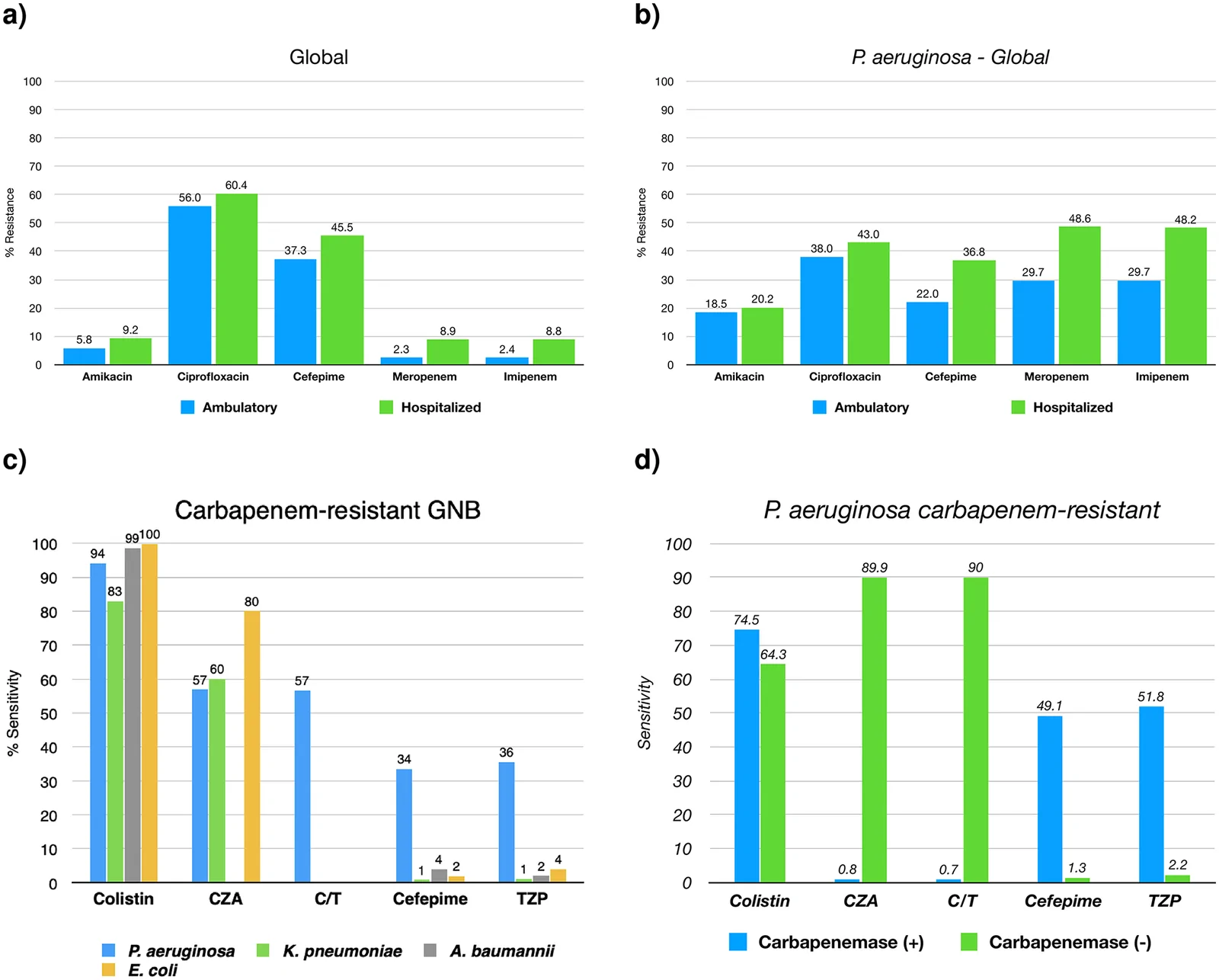
Antibiotic resistance and susceptibility profiles among ambulatory and hospitalized patients with Gram-negative bacterial isolates. a) Overall prevalence of antibiotic resistance among ambulatory and hospitalized patients. b) Prevalence of antibiotic resistance in *Pseudomonas aeruginosa* isolates from ambulatory and hospitalized patients. c) Antibiotic susceptibility profiles of carbapenem-resistant Gram-negative bacteria. d) Antibiotic susceptibility of carbapenem-resistant *Pseudomonas aeruginosa* according to carbapenemase production status. Abbreviations: DTR, difficult-to-treat resistance; CZA, ceftazidime-avibactam; C/T, ceftolozane-tazobactam; TZP, piperacillin-tazobactam.
