# Supplementary material for: Carbapenem resistance and susceptibility to Ceftazidima/avibactam and Ceftolozane/tazobactam in Pseudomonas aeruginosa and Enterobacterales in a reference laboratory in Lima, Peru: A cross-sectional study
Source: PLoS One. 2026 Aug 24;21(8):e0356004. doi: 10.1371/journal.pone.0356004 (PMC13502689; doi:10.1371/journal.pone.0356004)
Supplement: Supplementary file 1 [file pone.0356004.s001.docx]

**S1 Appendix. List of abbreviations**

| **Abbreviation** | **Definition** |
| --- | --- |
| AMR | Antimicrobial resistance |
| MDR-GNB | Multidrug-resistant Gram-negative bacteria |
| CRE | Carbapenem-resistant Enterobacterales |
| CP-CRE | Carbapenemase-producing carbapenem-resistant Enterobacterales |
| CRPA | Carbapenem-resistant *Pseudomonas aeruginosa* |
| NCP-CRPA | Non-carbapenemase-producing carbapenem-resistant *Pseudomonas aeruginosa* |
| CZA | Ceftazidime/avibactam |
| C/T | Ceftolozane/tazobactam |
| ATM-AVI | Aztreonam/avibactam |
| IMI-REL | Imipenem/relebactam |
| DTR | Difficult-to-treat resistance |
| MBL | Metallo-β-lactamase |
| KPC | *Klebsiella pneumoniae* carbapenemase |
| NDM | New Delhi metallo-β-lactamase |
| IMP | Imipenemase metallo-β-lactamase |
| VIM | Verona integron-encoded metallo-β-lactamase |
| OXA | Oxacillinase |
| ESBL | Extended-spectrum β-lactamase |
| CLSI | Clinical and Laboratory Standards Institute |
| EUCAST | European Committee on Antimicrobial Susceptibility Testing |
| MALDI-TOF | Matrix-assisted laser desorption ionization–time of flight |
| IQR | Interquartile range |
| PR | Prevalence ratio |
| OR | Odds ratio |
| CI | Confidence interval |
